# Recent advances in the design of RAR α and RAR β agonists as orally bioavailable drugs. A review

**DOI:** 10.1016/j.bmc.2020.115664

**Published:** 2020-10-15

**Authors:** Alan D. Borthwick, Maria B. Goncalves, Jonathan P.T. Corcoran

**Affiliations:** a DrugMolDesign, 15 Temple Grove, London NW11 7UA, UK; b Neuroscience Drug Discovery Unit, Wolfson Centre for Age-Related Diseases, Guy’s Campus, King’s College, London SE1 1UL, UK

**Keywords:** Retinoic acid receptor, Alpha agonist, Beta agonist, SAR, RAR586, AC-261066, C286, Nerve injury

## Abstract

Retinoic acid receptors (RARs) α, β, and γ are members of the nuclear receptor superfamily. Compounds which bind to and activate the RARs are termed retinoids which regulate a wide variety of biological processes such as vertebrate embryonic morphogenesis and organogenesis, cell growth arrest, differentiation, and apoptosis, as well as their disorders. Although many synthetic selective RARα, RARβ, and RARγ agonists have been designed and prepared, these have generally been lipophilic acids without good drug-like properties and with low oral bioavailability. Recently this has been changing and drug design approaches to highly potent and selective RARα and RARβ agonists with low lipophilicity that are orally bioavailable and less toxic have been developed, that have a range of potential therapeutic uses. This review covers these new advances.

## Graphical abstract

**Figure:**
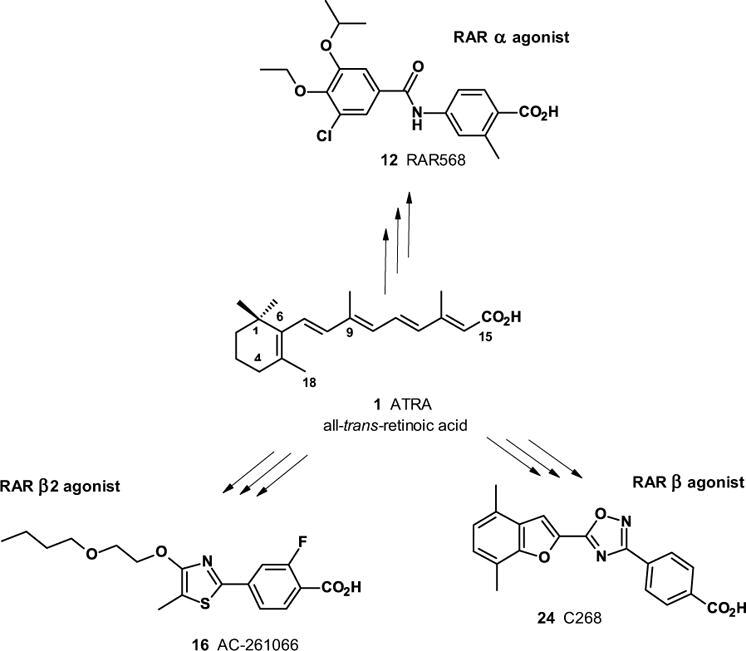

## 1 Introduction

There are three retinoic acid receptors (RARs) α, β, and γ which are members of the nuclear receptor superfamily.^1^ Compounds which bind to and activate the RARs are termed retinoids these include, all-trans-retinoic acid (ATRA) which is derived from vitamin A and synthetic analogs.^2^

In order to carry out their transcriptional action RARs heterodimerise with other members of the nuclear receptor superfamily the retinoid X receptors (RXRs) of which there are also three types α, β, and γ.^3^ This heterodimer binds to specific sequences in the DNA, the retinoic acid response elements (RAREs), once the retinoid binds transcription occurs.^1^ Further complexity occurs in the pathway due to the ability of the RXRs to bind to the nuclear orphan receptors, of which there are 48 human members.^3^

Retinoids regulate a wide variety of biological processes such as vertebrate embryonic morphogenesis and organogenesis, cell growth arrest, differentiation, and apoptosis, as well as their disorders.^4^

**Fig. 1:**
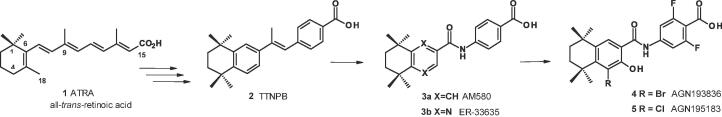
RARα agonists.

Synthetic RARα, RARβ, and RARγ agonists have been developed from the endogenous ligand all-trans-retinoic acid (ATRA), which is prone to double-bond isomerisation and to oxidation by metabolic enzymes. These synthetic retinoids are much more stable, as well as being more active and selective and usually consist of a lipophilic ring, a linker and a carboxylic acid (Fig. 1).

Although many synthetic selective RARα, RARβ, and RARγ agonists^5^, 6., 8 have been designed and prepared, these have generally been lipophilic acids without good drug-like properties and with low oral bioavailability. Recently this has been changing and drug design approaches to highly potent and selective RARα^9^ and RARβ agonists^10^, ^11^, ^12^^,^ with low lipophilicity that are orally bioavailable and less toxic have been developed that have a range of potential therapeutic uses. This review covers these new advances.

## 2 RARα agonists

### 2.1 Occurrence and activity

The RARα isoform is found in the majority of tissues and has been implicated in a number of diseases, most notably acute promyelocytic leukemia (APL). Selective RARα agonists have been shown to inhibit proliferation and induce apoptosis of mammary tumour oncogenesis in murine models (MMTV-neu and MMTVwnt1 transgenic mice) relevant to human cancer,^13^ and to inhibit LPS-induced B-lymphocyte proliferation.^14^ Selective RARα agonists have also been shown to prevent neuronal cell death caused by amyloid-β and, when administered orally, can prevent amyloid-β production and Alzheimer’s disease progression in a mouse model.^15^ It has been shown^16^ that selective RARα agonists suppressed allospecific immune response and significantly prolonged the survival of mouse cardiac allografts and can ameliorate nephritis in lupus-prone mice, NZB/NZW F1.^17^ This supports the rationale for using RARα agonists as immunosuppressants in human organ transplantation. Thus selective RARα agonists have the therapeutic potential for the treatment of cancer, dermatological diseases, Alzheimer’s disease and immunological disorders.

### 2.2 SAR development of RARα selectivity the amide linkage

One of the aims of the earliest modifications of the natural ligand ATRA **1** was to reduce the instability and flexibility of its polyene structure by replacing the diene/triene moieties with aromatic rings. This gave the tetrahydronaphthalene benzoic acid TTNPB **2** (Fig. 1) which had the same activity as ATRA **1** and behaved as a retinoic acid receptor pan-agonist with activity at RARα, RARβ, and RARγ in the co-transfection assay.^18^ However this highly lipophilic benzoic acid derivative TTNPB **2** was toxic.

In the search for subtype-selective agonists, one of the most successful modifications in the design of synthetic retinoids was to replace the double bond linker between the hydrophobic ring and carboxylic acid in TTNPB **2** with an amide linker to give AM580 **3a,**^5^ one of the first RAR subtype selective retinoids discovered that has higher affinity for the RARα subtype than for RARβ or RARγ (Table 4).

**Fig. 2:**
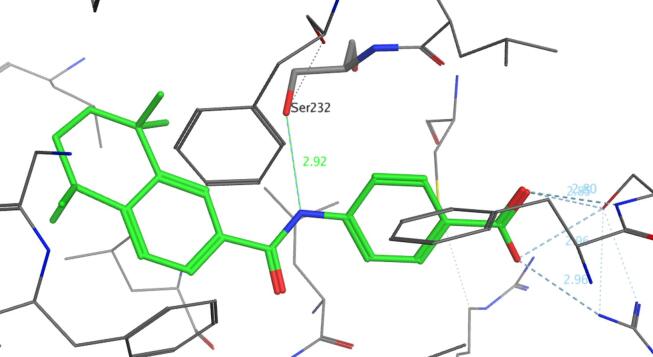
Crystal structure AM580 **3a** bound to RARα with key Ser232 interaction highlighted (PDB: 3KMR).^7^

This polar linker has several advantages, it lowers the lipophilicity (Δ Log P ≈ 2) and has the potential to form hydrogen-bonds in the ligand binding domain (LBD) of the various RARα, β and γ isoforms which could enhance selectivity. In fact it has been claimed that a strong hydrogen bond between the polar amide linkage in AM580 **3a** and residue Ser232 in LBD of RARα (Fig. 2),^7^ in contrast to lipophilic Ala225 and Ala234 residues present in the LBDs of RARβ and RARγ respectively, results in RARα selectivity.^6^ Selectivity can be further increased by incorporating substituent’s such as halogens on the hydrophobic group as well as by adding a fluorine substituent ortho to the carboxylic acid group (e.g., AGN193836 **4**, which was the first monospecific RARα retinoid to be synthesised).^19^

Although AM580 **3a** and AGN195183 **5** (Fig. 1) have moderate and good selectivity respectively for RARα, over RARβ, and RARγ they are still quite lipophilic (cLog P 6.3 and 7.2), without any significant oral bioavailability. In addition AM580 **3a** has been shown to be toxic,^20^, ^21^ and the more recently discovered compound AGN195183 **5**^22^ which was in Phase I clinical trials for cancer has also been discontinued.^23^

### 2.3 RAR568 (**12**) a novel RARα agonist template with lower lipophilicity

#### 2.3.1 Discovery of the initial hit 3,5-dichloro-4-ethoxy derivative **7**

The initial aim to reduce lipophilicity was to find a novel, potent, highly selective RARα agonist not based on the bicyclic 5,5,8,8-tetramethyl-5,6,7,8-tetrahydronaphthalene class present in **3a, 4** and **5** (Fig. 1) that was ligand efficient, orally bioavailable and without the lipophilic obesity seen with **3a, 4** and **5**, that could be developed into a novel oral therapeutic.

**Fig. 3:**
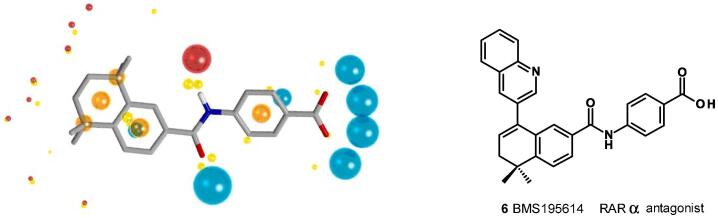
Cresset FieldScreen representation of bioactive conformation of AM580 **3a**.^a^. ^a^Blue field points (spheres) highlight energy minima for a positively charged probe, red for a negative probe. Yellow spheres represent attractive van der Waals minima for a neutral probe and orange spheres represent hydrophobic centroids. Oxygen atoms are shown in red, nitrogen in blue. The size of the points is related to the strength of the interaction.

A ligand-based virtual screening approach was used where the crystal structure of the selective RARα antagonist BMS195614 **6** in the human RARα active site,^24^ was overlaid with AM580 **3a**, the antagonist removed and the resulting complete assembly minimized to give the putative bioactive conformation of agonist AM580 **3a**. This procedure was also performed for AGN193836 **4** to get its bioactive conformation. Molecular fields were added to each of these bioactive conformations (Fig. 3) and then these were used to search the Cresset’s database of 2.5 M commercially available molecules.

The 200 compounds that had the highest field overlays, Lipinski likeness, and synthetic tractability, were purchased. These were tested in transactivation assays at the RARα, β and γ receptors.^9^

This produced several potent hits, including the lead compound 4-(3,5-dichloro-4-ethoxybenzamido) benzoic acid **7** (Table 1). This was much less lipophilic (cLog P = 4.4) than AGN195183 **5** (cLog P = 7.2) yet still had moderate RARα agonist potency and good selectivity over the RARβ and RARγ receptors. In addition, **7** was found not to be cytotoxic in COS-7 cells and it had no systematic Cyp450 liability. The non-alkyl 3,4,5 tri-substituted (phenylcarboxamido) benzoic acid template represented by **7** is a novel class of retinoids.

**Table 1:** Potency and Selectivity of 3,5-dichloro-4-ethoxy RAR α agonist **7** and AGN195183 **5**.^9^

**Figure:**
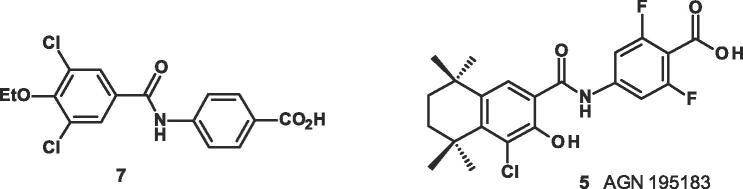

	Subtype-specific transactivation^a^ Relative EC_50_^b^					
compd	RAR*α*	RAR*β*	*β*/*α* ratio c	RAR*γ*	*γ*/*α* ratio c	cLogP e
**5**	11	1564	141	9836	867	7.2
**7**	24	1917	79	>300000	>12,500	4.4
**ATRA**	1.0 (1.51 nM)^d^	1.0 (1.52 nM)^d^		1.0 (0.2 nM) d		

a Transactivation assays for the RAR alpha, beta and gamma receptors were performed using each of the mouse RAR ligand binding domains, Subtype-specific activity is expressed in terms of relative EC_50_ which is the concentration of retinoid required to produce 50% of the maximal observed response, normalised relative to that of ATRA.

b Mean EC_50_ for each compound divided by the mean EC_50_ of ATRA. Values were obtained from three separate experiments. Errors in these assays are approximately 20% of the mean values.

c The relative EC_50_ ratios of *α* to *β* and *α* to *γ*.

d Mean of ATRA EC_50_ (nM).

e cLog P values were calculated in ChemDraw.

The aim with this novel template was to increase the RARα potency and selectivity over RARβ while retaining the excellent selectivity over RARγ shown by **7** and achieve oral bioavailability in the rat.

#### 2.3.2 SAR to optimise the RARα agonist selectivity and oral bioavailability to give RAR568 (**12)** (see Fig. 4)

Initial SAR showed that the three aromatic substituents in **7** seemed important for potency as the disubstituted, 3,5-dichloro derivative without the 4-ethoxy group was less potent at RARα and also less selective than the 3,5-dichloro-4-ethoxy derivative **7** at RARβ and RARγ. This helped focus the SAR on derivatives with a 3,4,5 substituted aromatic ring.

**Fig. 4:**
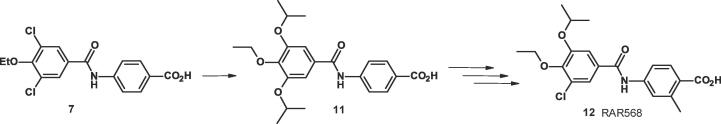
Lead optimisation.

Modification of the size or length of the 4-alkoxy substituent in **7** lost RARα potency or selectivity at RARβ and RARγ. These compounds also had a high mouse, and moderate human intrinsic clearance, and generally their PK profile was poor.

However a patent analysis of this class of compounds showed that non-alkyl substituents in the 3,4,5-substituted aromatic ring of **7** appeared novel. The medicinal chemistry parameters of the non-alkyl 3,5-substituents of the initial 4-OEt derivatives **7**, **8**, **9,** and 3,5-dialkyl substituents **10** were investigated (Table 2). Ranking these four derivatives in terms of RARα potency against the properties of the 3,5 substituents in the second aromatic ring, such as size (MR), lipophilicity (**π**) and electronic resonance (σ) (Table 2), showed that potency only increases with the lipophilicity (**π**) of the 3,5-sustituents (and not with the size or resonance effects of these substituents).

**Table 2:** 3,5-Disubstituted-4-ethoxy derivatives.

**Figure:**
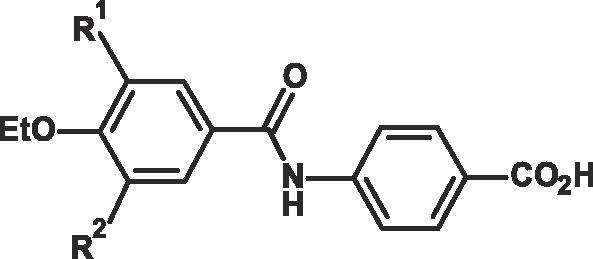

compd	R^1^	R^2^	MR^a^ R^1^ + R^2^	π^b^ R^1^ + R^2^	σ^c^ R^1^ + R^2^	RAR*α* rel EC_50_^d^
**8**	EtO	EtO	25	0.76	0.2	370
**7**	Cl	Cl	12.06	1.42	0.74	24
**9**	Br	Br	17.76	1.72	0.78	5
**10**	*^t^*Bu	*^t^*Bu	39.24	3.96	−0.20	0.2
**11**	^i^PrO	^i^PrO	34.12	1.70	0.20	26^e^

a Sum of size (MR) of *meta* substituents R^1^ and R^2^.

b Sum of lipophilicity (π) of substituents R^1^ and R^2^.

c Sum of electronic resonance effect (σ) of *meta* substituents R^1^ and R^2^, for parameters see ref 9.

d relative EC_50_ see **^a,b^**Table 1

e partial agonist see **^e^**Table 3

A search of possible aromatic substituents showed that the isopropoxy group has a similar lipophilicity to a chlorine/bromine atom found in **7/9** and a similar size to a *tert*-butyl group found in the more potent derivative **10.** This suggested that the 3,5-diisopropoxy-derivative **11** should be at least as active as the chloro- and bromo-derivatives **7** and **9**, and also why the 3,5-diethoxy analog **8** which is the least lipophilic, is the least active.

Encouragingly **11** proved to have good RARα potency and high selectivity over RARβ and RARγ (Table 3). It also had low mouse and human intrinsic clearance with excellent oral absorption and bioavailability (81%) in the rat, although it was shown to be only a partial RARα agonist. The close profile of **7** and **11** in terms of RARα potency, as well as RARβ and RARγ selectivity, shows that in this case, the *i*PrO group is a good bioisostere of the Cl group. This led the project away from the 3,5-dichloro template and enabled exploration of the alkoxy derivatives at these positions which give a lipophilic surface without the high lipophilicity of the similar sized tertiary butyl group seen in **10**, making the template more drug-like.

**Table 3:** Comparison of the RARα Agonist Potency, and selectivity versus the RARβ and RARγ Receptors, Intrinsic Clearance and Pharmacokinetic Profile in Rat for **7** and **11**.^9^

compd	RAR*α* rel EC_50_	*β*/*α* ratio	*γ*/*α* ratio	LogD^a^ pH 7.4	LE^b^	intrinsic Cl_int_^c^	rat PK^d^		
						Mouse/human	AUC po	Cl	*F*%
**7**	24	79	>12,500	1.7	0.45	127/18	–	–	–
**11**	26^e^	175	2190	1.6	0.36	8/4	783,782	1	81

a Measured by octanol/buffer shake flask method at pH 7.4.

b LE values were calculated by LE =  (RT ln K_d_)/N, presuming EC_50_ ≈ K_d_.**^9^**

c Intrinsic clearance Cl_int_ data for screening purposes only: Mouse and Human microsomes were incubated with the test compound at 37 °C in the presence of the co-factor, NADPH. The data is the mean of 5 separate experiments. Compound disappearance monitored over 45 min period. SEM is <10% of the mean values.

d Rat PK (n = 4): AUC (ng·min mL^−1^) at 10 mg/kg, 8% Ethanol/92% PEG-400 formulation, Cl in mL min^−1^ fkg^−1^.^9^ ND = not determined.

e Compound behaves as a partial agonist relative to the amplitude of the normalizing ATRA output. Where partial agonism is defined for a compound which has a maximum activation of <80 percent of the ATRA max activation on the same plate, on more than one occasion in 3 independent assays.

**Table 4:** Comparison of the RARα Agonist Potency, selectivity versus the RARβ and RARγ Human and Mouse Receptors, Human Intrinsic Clearance and Pharmacokinetic Profile in Rat for RAR568 **12** and AM580 **3a**.^9^

compd	RAR*α* rel IC_50_^a^	RAR*α* rel EC_50_^b^m/chu	*β*/*α* ratio^b^m/chu	*γ*/*α* ratio^b^m/chu	intrinsic Cl_int_^d^	rat PK^e^			fLog D pH 7.4
					human	AUC po	Cl	*F*%	
RAR568 **12**	3.6	1.6/0.6	200/298	11,000/>13000	5.3	70,765	7	40	1.8
AM580 **3a**	9	0.02/0.13	1130/162	826/505	15.6	–	–	–	2.8

a RAR*α* binding assay. The relative IC_50_ is the mean IC_50_ for each compound divided by the mean IC_50_ of ATRA (IC_50_ = 0.6 nM). Values were obtained from three separate experiments.

b m = mouse receptor, see Table 1.

c hu = human receptor.

d Human microsomes Cl_int_ (µL/min/mg protein),

e AUC po ng·min mL^−1^. Cl mL/kg/min.

f Log D see Table 3.

Further analogs of this trialkoxy template **11** were investigated in an attempt to increase its alpha potency while maintaining the excellent beta and gamma selectivity as well as its good PK profile. Increasing the size of the 3,5-substituents or the 4-substituents in **11** maintained the good RARα potency and RARβ selectivity but lost selectivity against RARγ. In contrast, it was found that moving to the 4′-(3-chloro-4,5-dialkoxybenzamido) benzoic acid class of derivatives gave an increase in RARα potency, often however producing partial agonists.

It had been shown from substitution at the ortho-position of benzoic acid **7**, with a range of groups that methyl groups are the best at increasing potency while maintaining good RARβ and RARγ selectivity. This led to methyl substitution at the ortho-position of the 4′-benzoic acid ring of a series of 4′-(3-chloro-4,5-dialkoxybenzamido) benzoic acid derivatives which gave a series of full agonists, the best of which was the novel RARα agonist 4-(3-chloro-4-ethoxy-5-isopropoxybenzamido)-2-methylbenzoic acid **12** (RAR568) in terms of RARα agonist potency and selectivity versus RARβ (2 orders of magnitude) and RARγ (4 orders of magnitude) at both the human and mouse RAR receptors (Table 4). Predevelopment studies showed that this potent RARα-specific agonist with improved physicochemical properties has high bioavailability (>80%) in both mice and dogs with a good PK profile and drug-like properties and it was also shown to be negative in the cytotoxicity and genotoxicity screens warranting further consideration as a potential therapeutic agent.^25^

Recently it was shown that RAR568 **12** treated regulatory T cells derived from patients with Crohn's disease retain optimal suppressive ability and phenotypic stability compared with standard culture conditions. ^26^

## 3 RARβ agonists

### 3.1 Introduction: BioTargets

RARβ regulates essential pathways associated with the tumour-suppressive effects of retinoids in various epithelial cells and it has been suggested that RARβ signalling may act as a potential tumour suppressor.^27^ It has also been shown that RARβ agonists have the potential to be of use in the treatment of nerve injury. RARβ agonists can activate the RARβ receptor which initiates axonal outgrowth in models of nerve injury and leads to functional recovery.^28^ It is a conserved pathway both with regard to different types of nerve injury, including optic nerve, diabetic neuropathy, avulsion and spinal cord injury and between species as it is required for mammalian and amphibian nerve regeneration.

It has been claimed that rational drug design based on the current crystal structures of RAR subtypes is likely to be inefficient for discovering RARβ subtype selective ligands as the ligand-binding domain (LBD) of RARβ subtype only differs by one residue from that of its RARα analogue and by two residues from that of its RARγ analogue.^29^ Ligands that display selectively for RARα or RARγ have been explained on the basis of specific hydrogen bonds formed with the polar Ser^232^ (H3) ^30^ and the weakly polar Met^272^ (H5) ^31^ in RARα and RARγ, respectively. However, no such discriminatory bond can be established in RARβ LBP so the development of RARβ-selective ligands is more challenging ^32^ and requires alternative strategies.

### 3.2 AC-261066 (**16**) a selective RARβ2 agonist

The RARβ subtype consists of five known isoforms (β1–β5) and finding RARβ isoform-selective ligands is a challenge since the ligand binding domains of the isoforms are identical. The variation between the RARβ1 and RARβ2 isoforms is for example located within the proximal *N*-terminus, which encompasses the ligand-independent activation domain (AF-1).^33^ However, Lund et al discovered the RARβ2 selective agonist 4′-Octyl-4-biphenylcarboxylic acid, AC-55649 **13** by HTS screening a 160,000 small molecule library against the RAR β2 receptor in a functional mammalian cell-based R-SAT assay.^10^ Although this agonist showed transcriptional potency of (pEC_50_ 6.9, 92% eff) and 100-fold selectivity vs the other RARs it is a highly lipophilic (Log P = 7.7) biphenyl carboxylic acid with very low aqueous solubility (<0.001 mg/mL) Table 5.

**Table 5:** Activity and Solubility of RARβ2 Agonists.

**Figure:**
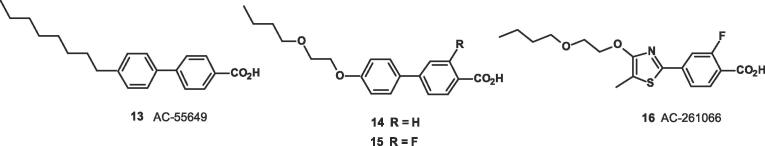

			RARβ2^a^	
compd	cLogP	Aq. Soly^b^	Eff (%)	pEC_50_
**13**	7.7	<0.001	92 (±24)	6.9
**14**	~4.7	–	78 (±19)	7.2
**15**	4.6	0.02	108 (±5)	7.7
**16**	5.2	4.8	106 (±26)	8.1

a pEC_50_ and efficacy values are the mean values of at least three experiments ± SD, with AM580 used as reference and set to 100% Eff.

b mg/mL, the solubility was measured in phosphate buffer, pH 7.4.

To improve on its potency and lack of aqueous solubility a lead optimisation programme was started that involved reducing the overall lipophilicity by replacing the 4′-alkyl group with a range of 4′-alkoxy groups and replacing the phenyl ring with heterocyclic rings. Replacing the 4′-octyl group (in **13**) with 4′-butoxyethoxy group gave **14** and an increase in potency pEC_50_ 6.9 to 7.2 and a big drop in lipophilicity (ΔlogP ~ 3). Adding an ortho 2F group to the acid in **14** gave **15** and a further increase in potency pEC_50_ 7.2 to 7.7, which was also ca.20 fold more soluble than **13**. But the biggest increase in aqueous solubility was achieved by replacing the second phenyl ring with a thiazole to give AC-261066 **16** which was 240 fold more soluble than **15** which also had an increase in potency pEC_50_ 7.7 to 8.1 (Table 5). In addition **16** had promising drug–like properties including a 52% bioavailability but with a clearance of 41 mL/(min·kg) in the rat.^11^

AC-261066 **16** has been shown to possess anti-diabetic properties,^34^ reduce the hepatic stellate cell activation in non-alcoholic fatty liver disease.^35^ and exert cardioprotective effects in mice.^36^

### 3.3 RARβ agonist C286 (**24**)

It has been shown^37^ that stimulating the retinoid signalling pathway in animal models of nerve injury leads to axonal outgrowth and functional recovery, and that RARβ signalling is required for retinoid mediated neurite outgrowth of neurons.^38^ In contrast, signalling by RARα, RARγ or the RXRs has no effect on this action. In addition it has been shown^28^ that the RARβ agonist, CD2019 **25**, can activate the RARβ receptor in a dose dependent manner. This initiates axonal outgrowth in models of nerve injury and leads to functional recovery. However CD2019 **25** is a highly lipophilic compound that is not significantly orally bioavailable and shows only weak to moderate selectivity over RARα and RARγ receptors. AC-261066 **16**,^11^ more recently described as a selective RARβ agonist is less potent than CD 2019 **25** and less selective than the latter over RARα (Table 8). The aim was to identify a more drug-like, highly potent and selective RARβ agonist that was orally bioavailable and which had the potential to be useful in the treatment of nerve injury.

#### 3.3.1 Lead discovery, replacing the amide linkage

To obtain a RARβ selective agonist the amide linker in the selective RARα agonist **7** obtained from a ligand-based virtual screening programme, was replaced with a series of 5 membered heterocyclic rings. Kikuchi et al. ^39^ replaced the amide linkage in AM580 **3a** and its quinoxaline analogue ER-33635 **3b** with 5 membered heterocyclic rings and showed that the 2,5-pyrrole linker gave RARα selective agonists. The best heterocyclic linkage in terms of RARα agonist potency in the transfection assay was 2,5-pyrrole > 2,4-furan > 2,4-thiophene > 3,5-pyrazole > 3,5-furan > 2,4-thiazole > 2,4-pyrrole > 2,5-thiophene > NMe-2,5pyrrole > 2,5-imidazole. However, apart from the 2,5-pyrrole, the rest of the heterocyclic ring derivatives were slightly more potent at RARβ than RARα. With this selective Beta precedent in mind, the replacement of the amide linkage in **7** with a variety of 5-membered heterocycles (Table 6) was investigated.^12^

**Table 6:** Heterocyclic derivatives in RAR α, β and γ transactivation assays.^12^

**Figure:**
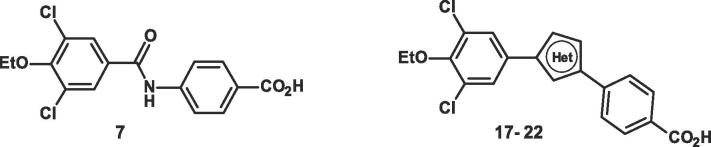

cpd	Het	α EC_50_ nM^a^	β EC_50_ nM^a^	Fold Selectivity for β over α^b^	γ EC_50_ nM^a^	Fold Selectivity for β over γ^b^	cLogP^d^
**ATRA**		1.9	1.2	1.56	0.9	0.75	
**7**		46	1227	0.037	30,000	24	4.4
**17**		240^c^	120	2	160	1.3	6.1
**18**		594^c^	423	1.4	ND	–	5.6
**19**		60	28	2.1	45	1.6	5.5
**20**		18^c^	1.5	12	28	19	5.1
**21**		31	110	0.28	5.4	0.05	5.1
**22**		58	63	0.92	150	2.4	4.3

a Transactivation assays for the RAR alpha, beta and gamma receptors were performed using each of the mouse RAR ligand binding domains. Values usually obtained from three separate experiments. Errors in these assays are approximately 20% of the mean values. Transactivation Assays details see supplementary data, reference 12 and reference 9. ATRA is all trans retinoic acid.

b The EC_50_ ratios of α to β and γ to β.

c Compound behaves as a partial agonist relative to the amplitude of the normalising ATRA output.

d Ref. 9.

Changing the amide linkage in **7** to thiazole and imidazole gave derivatives **17** and **18** that were weakly active as RARα agonists, but were more potent than amide **7** as RARβ agonists, although only weakly selective for RARβ vs RARα. The oxazole **19** was >40-fold more potent than **7** as an RARβ agonist and had similar agonist potency for all three subtypes.

Surprisingly however, increasing the number of heteroatoms in the heterocyclic ring to give the oxadiazole **20** resulted in a highly potent RARβ agonist and that had 12- and 19-fold selectivity as an agonist over RARα and RARγ respectively. This RARβ agonist selectivity and potency was lost when the isomeric 1,2,4-oxadiazol-5-yl benzoic acid derivative **21** and the 1,3,4-oxadiazol-2-yl benzoic acid compound **22** were examined (Table 6).

#### 3.3.2 Lead optimisation

Replacements for the 3,5-dichloro-4-ethoxyphenyl ring in **20** with other heterocyclic and aryl rings found in known RAR agonists were also investigated (Table 7).

**Table 7:** 1,2,4-oxadiazol-3-yl benzoic acid derivatives in RAR α, β and γ transactivation assays.^12^

**Figure:**
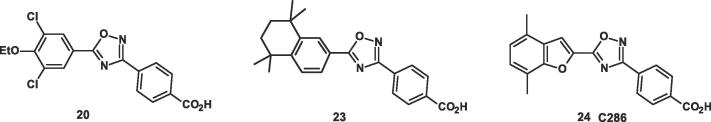

cpd	β EC_50_ nM^a^	α EC_50_ nM^a^	γ EC_50_ nM^a^	Fold Selectivity for β over α^b^	Fold Selectivity for β over γ^b^	cLogP ^d^
**1** ATRA	1.9	1.2	0.9	0.6	0.5	–
**20**	1.5	18^c^	28	12	19	5.1
**23**	4200	18	17	0.0043	0.0041	7.2
**24**	1.9	26	11	13	5.6	5.3

^a, b, c, d^ See Table 6.

**Table 8:** Selective RARβ agonists.^12^

**Figure:**
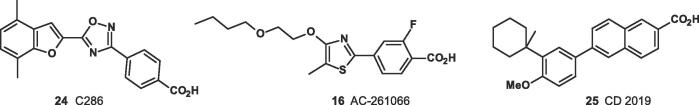

cpd	β EC_50_ nM^a^	α EC_50_ nM^a^	γ EC_50_ nM^a^	Fold Selectivity for β over α^b^	Fold Selectivity for β over γ^b^	Cl mL/kg/min rat/dog	F% rat/dog	cLogP^e^
C286 **24**	1.9	26	11	13	5.6	3.7/1.1	80^c^/45^d^	5.3
AC-261066 **16**	12	70	33	5.8	2.8	41^e^/--	52^f^/--	4.9
CD2019 **25**	0.83	9.2	1.6	11	1.9	–	–	8.0

^a, b, e^ See Table 6. ^c^ iv dose 0.5 mg/kg administered in 4% DMSO, 38% PEG-400, 58% (0.9%) NaCl. Oral doses of 1, 3 and 10 mg/kg prepared in 8% ethanol and 92% PEG-400. ^d^ iv dose 0.5 mg/kg administered in 2% DMSO, 98% aqueous hydroxypropyl-β-cyclodextrin (22.5% w/v). Oral dose 3 mg/kg administered in 3% DMSO, 97% aqueous hydroxypropyl-β-cyclodextrin (22.5% w/v). For assay description ^c,d^ see Ref. 12. For ^e,f^ see Ref. 11.

Relative to **20**, the 5,5,8,8-tetramethyl-5,6,7,8-tetrahydronaphthalene ring derivative **23** lost >2700 fold in potency as a RARβ agonist whilst retaining most of its potency at RARα. In contrast, the 4,7-dimethylbenzofuran derivative **24** maintained a similar potency and selectivity profile to **20** and with the desire to move away from the dichlorophenyl motif, this became the lead compound. Changing the 4,7-dimethylbenzofuran ring in **24** to the benzothiophene or the benzoxazole analogues or even the 4-trifluoromethyl-7-fluorobenzofuran derivative lost RARβ agonist potency and RARβ/RARα selectivity. Also substitutions in the benzoic acid ring of **24** to give the 2-methyl, 3-methyl, 2-fluoro or 3-fluoro derivatives resulted in a loss of potency or RARβ selectivity when compared to **24**.^12^

The lead RAR β agonist C286 **24** has a high potency at RAR β (similar potency to ATRA) and behaves as a full agonist. It has a selectivity for RARβ over RARα of 13-fold, while selectivity for RARβ over RARγ is 5.6-fold. Comparison of **24** with the selective RARβ agonist AC-261066 **16** ^11^ (Table 8) showed that **24** is a more potent and selective RARβ agonist. Whilst compound **24** is marginally less potent than CD2019 **25**, it has a better selectivity for RARβ over RARα and RARγ and is over two orders of magnitude less lipophilic. Also **24** had a similar RARβ potency (EC_50_ = 2.05 nM) and selectivity for RARβ over RARα (23 fold) and RARγ (5 fold) against the human RAR ligand-binding domains in a luciferase transactivation assay.^12^

The more drug-like template present in **24** (Table 9) translates into a good *in vitro* and *in vivo* profile for this RARβ agonist. Compound C286 **24**, possesses favourable physicochemical properties: water soluble (>100 μM as the sodium salt), good permeability, it was not a PGP substrate, with no significant inhibition IC50 > 25 μM against five Cyp450 isozymes, has a human and mouse plasma protein binding of 98% and 95% respectively and a very high stability in human microsomes. Compound **24** was also found to possess a promising pharmacokinetic profile in both rat and dog, with a low rate of blood clearance, a moderate half-life and a good oral bioavailability of >44% in both species (Table 8). It was also found to penetrate the CNS, with nearly equivalent amounts detected in brain tissue when compared to plasma, 8 h after dosing orally to rats. Furthermore, it has been shown to be inactive in cytotoxicity and genotoxicity *in-vitro* screens. The no adverse effect level (NOEL) in rat was found to be 1 mg/kg and this was due to defects in bone plate closure as these grow continuously in rats, whereas in Beagle the NOEL was higher at 3 mg/kg as the bone plates are not affected in adult dogs.^12^ These doses have been shown to effective in stimulating axonal outgrowth in nerve injured rats.^12^

**Table 9:** Physico-chemical and *in vitro* properties of RARβ agonist C286 **24.**

LogD^a^ 7.4	Solubility^b^ µM pH 7.4	MDCK^c^ Papp ×10^-6^ cm/s	MDCK^c^ asymmetry ratio	Cyp450^d^ IC_50_ μM	Human Cl_int_^e^ µL/min/mg protein
2.8	>100	28	0.8	>25	<1

For ^a, b, c, d, e^ see reference 12. ^d^ Cyp450 isoforms 1A2, 2C9, 2C19, 2D6, 3A4.

#### 3.3.3 Structural rationale for the selectivity of oxadiazoles **20** and **21.**

The bioisosteric oxadiazole isomers are known to have significant differences in their various physical and pharmaceutical properties due to variation in hydrogen bond acceptor and donor strength.^40^, ^41^ The N-2 hydrogen bond acceptor in the 1,2,4-oxadiazol-5-yl isomer **21** being in different position to the N-2 hydrogen bond acceptor in the 1,2,4-oxadiazol-3-yl isomer **20** when these are bound in the LBD’s of RARβ and RARα which may account for their difference in activity.

While molecular docking of these molecules gives a clear assessment of the binding energies and complementarities of the ligands to the three proteins RARα, RARβ, and RARγ we were unable to find a suitable explanation of the changes in agonist**/**partial agonist behaviour as described in this paper using the Cresset software Flare. It may be that some dynamic reorganisation or accommodation of water is needed to fully explain the data.^42^

#### 3.3.4 Bioactivity of C286 (**24**)

##### 3.3.4.1 Treatment of nerve injury

For successful axonal regeneration to occur numerous pathways need to be activated and this has impeded successful drug development given that many drugs only target one pathway. The pathways for successful functional recovery include axonal outgrowth, modulation of the glial scar from growth inhibitory to growth permissiveness, correct pathfinding synaptogenesis and myelination. An ideal drug would be one that can stimulate all these pathways. RARβ is upregulated in the neurons after injury but requires ligand binding activation to sustain its expression levels and elicit a biological response.^43^, ^44^ Functional recovery has been demonstrated with the RARβ agonist C286 **24** which increase neurite outgrowth *in vitro* and induce sensory axon regrowth *in vivo* in a rodent model of avulsion and crush injury,^12^ and thus has the potential to be a therapeutic agent for the treatment of nerve injury.

Recent work with C286 **24** has shown the multifactorial nature of RARβ signalling in axonal/neurite outgrowth. In a model of dorsal root avulsion where the sensory nerve is cut and implanted into the spinal cord, the inhibitory scar tissue which arises is reorganised into a growth permissive environment. It achieves this in a number of ways. The astrocyte which make up the scar become organised into tunnels through which the regenerating axons can grow.^44^ One of the mechanisms involved in this is the secretion of neuronal Phosphatase tensin homolog (PTEN) in exosomes which prevents the proliferation of the astrocytes forming the scar.^44^ In addition, cells expressing Neuron-Glia 2 (NG2 cells) which are often perceived as having a axonal growth inhibitory effect, in response to C286 **24** act as pathfinders to the growing axon and then differentiate into myelinating oligodendrocytes.^45^ They achieve this by expressing the retinaldehyde dehydrogenase 2 (RALDH2) enzyme which synthesises retinoic acid (RA). The precursor of RA, retinal is induced by C286 **24** in the neurons by stimulating the expression of alcohol dehydrogenase (ADH) IV, the retinal is then transferred across to the NG2 cells which is then converted into RA. The RA synthesised by the NG2 is taken up by the growing axon for further growth. Thus the localised production of RA attracts the growing axon and induces a positive feedback loop suggesting that the C286 **24** is only required for a short time to initiate the regeneration pathways.

Much work has been spent on understanding the inhibitory environment, these factors include the inhibitory extracellular matrix molecules, the condroitinase proteoglycans (CSPGs) and it has been shown that chondroitinases can have a positive effect on axonal outgrowth. Recent work has shown that C286 **24** can modulate the expression of CSPGs by neuronal secretion of decorin which promotes myelination and aids axonal growth. In addition to this the role of C286 **24** in myelination related to its CSPG modulation has been uncovered. The decorin a potent scavenger of CSPGs causes a decrease in calcium in the NG2 cells^44^, ^46^ which prevent the secretion of RA in exosomes, the RA is therefore retained in the NG2 cells which activate RARα, which induces myelination.^44^, ^46^

##### 3.3.4.2 Prevention of neuropathic pain

The RARβ agonist drug C286 **24** also demonstrates efficacy in a pre-clinical neuropathic pain (NP) model restoring multiple pathways via DNA repair mechanisms.^47^

Neuropathic pain (NP) is associated with profound gene expression alterations within the nociceptive system and is a common comorbidity of spinal cord injuries. DNA mechanisms, such as epigenetic remodeling and repair pathways have been implicated in NP. Using a rat model of peripheral nerve injury it has been found that a 4-week treatment with C286 **24** initiated 2 days after the injury normalised pain sensation.^47^ Genome-wide and pathway enrichment analysis showed that multiple mechanisms persistently altered in the spinal cord were restored to preinjury levels by the agonist.^47^

This illustrates the multifactorial nature of C286 **24** in that it can modulate many pathways and gets away from the dogma that by targeting one pathway will have therapeutic benefit.

## 4 Conclusions

The major obstacle to the development of orally bioavailable RAR agonists has been the high lipophilicity of the natural carboxylic acid ligand and of the early synthetic agonists. This problem has been overcome by careful lead optimisation of a novel lead obtained from a ligand based virtual screening programme, which gave the highly potent and selective RARα agonist RAR586 **12** with high oral bioavailability and a good PK profile. A key element of this success was incorporation of heterocyclic linkers culminating in the discovery of the selective RAR beta agonist C286 **24** (logD, 2.8) showing high solubility and good oral pharmacokinetics. C286 **24** is currently in Phase I clinical trials (ISRCTN12424734) the results of which will be presented in due course.

## Declaration of Competing Interest

The authors declare that they have no known competing financial interests or personal relationships that could have appeared to influence the work reported in this paper.
